# Calcium influx through TRP channels induced by short-lived reactive species in plasma-irradiated solution

**DOI:** 10.1038/srep25728

**Published:** 2016-05-12

**Authors:** Shota Sasaki, Makoto Kanzaki, Toshiro Kaneko

**Affiliations:** 1 Department of Electronic Engineering, Tohoku University, 6-6-05 Aoba, Aramaki, Aoba-ku, Sendai 980-8579, Japan; 2 Graduate School of Biomedical Engineering, Tohoku University, 6-6-04 Aoba, Aramaki, Aoba-ku, Sendai 980-8579, Japan

## Abstract

Non-equilibrium helium atmospheric-pressure plasma (He-APP), which allows for a strong non-equilibrium chemical reaction of O_2_ and N_2_ in ambient air, uniquely produces multiple extremely reactive products, such as reactive oxygen species (ROS), in plasma-irradiated solution. We herein show that relatively short-lived unclassified reactive species (i.e., deactivated within approximately 10 min) generated by the He-APP irradiation can trigger physiologically relevant Ca^2+^ influx through ruthenium red- and SKF 96365-sensitive Ca^2+^-permeable channel(s), possibly transient receptor potential channel family member(s). Our results provide novel insight into understanding of the interactions between cells and plasmas and the mechanism by which cells detect plasma-induced chemically reactive species, in addition to facilitating development of plasma applications in medicine.

Plasma medicine is a rapidly emerging field, and a number of researchers have reported innovative applications of non-equilibrium atmospheric-pressure plasma (APP)^1^,^2^,^3^,^4^, including its use in the selective killing of cancer cells^5^, in blood coagulation for minimally invasive surgery, in assisting wound healing and tissue regeneration^6^, and as a gene transfer tool^7^,^8^,^9^. Non-equilibrium APP has a higher electron temperature (~several eV) than gas (ion) temperature^10^. This allows for strong non-equilibrium chemical reactions, and computer simulations and experimental results have shown that numerous chemically reactive species are generated in the plasma or in plasma-irradiated solution as a result of these specific reactions^11^,^12^,^13^,^14^,^15^,^16^,^17^. The fact that chemically reactive species (e.g., reactive oxygen species [ROS] and reactive nitrogen species [RNS]) are key components of APP in the plasma treatment of cells or living tissue is now widely accepted^1^,^2^,^3^,^4^. However, it remains unclear how cells detect the chemically reactive species and what types of chemically reactive species contribute to plasma-induced cellular responses.

Our research focuses on cytoplasmic calcium ions (Ca^2+^), which play key roles in many cellular processes, such as endocytosis, exocytosis, intermediate metabolism, neurotransmission, muscle contraction, cell motility, and cell division^18^. The cytosolic free calcium concentration ([Ca^2+^]_i_) in mammalian cells is normally maintained at extremely low levels (~100 nM) compared with the extracellular calcium concentration ([Ca^2+^]_i_ ~1 mM) and calcium concentration in the endoplasmic reticulum (ER) as an intracellular Ca^2+^ store. Temporary opening of calcium ion channels in the cell membrane or the ER membrane leads to a sharp rise in [Ca^2+^]_i_, resulting in the activation of various signal transduction pathways that regulate cell function. Some of these ion channels can open and close in response to extracellular mechanical, electrical, and chemical stimuli^19^,^20^,^21^. However, there are no reports throughoutly investigating the effect of plasma-produced species on calcium ion channels.

In this study, we investigated the interaction between intracellular calcium and chemically reactive species generated in plasma-irradiated solution in 3T3-L1 mouse fibroblasts, particularly with respect to calcium ion influx through transient receptor potential (TRP) channels triggered by the plasma-produced reactive species. Numerous previous reports suggest that biological responses in cells exposed to plasma-irradiated solution are mediated through calcium signaling cascades. The results of the present study therefore enhance understanding of the interactions between cells and plasmas and the mechanism by which cells detect plasma-induced chemically reactive species, in addition to facilitating development of plasma applications in medicine.

## Experimental

### Helium atmospheric-pressure plasma (He-APP) irradiation and calcium live-imaging system

**Figure 1:**
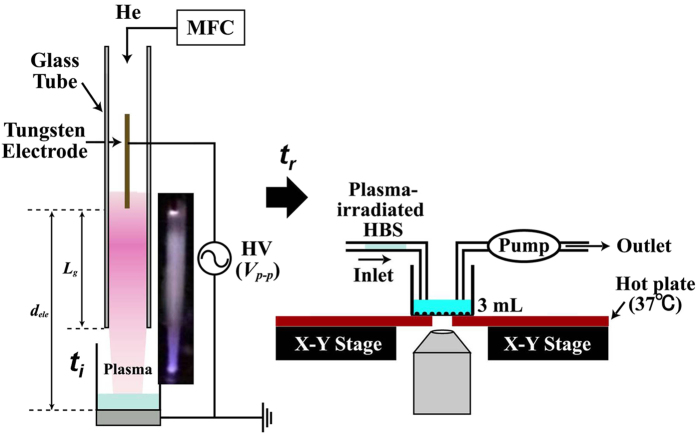
Schematic illustration of the experimental setup for the helium atmospheric-pressure plasma (He-APP) irradiation system and live-imaging of [Ca^2+^]_i_ after injection of He-APP–irradiated solution. The parameter *t*_*i*_ is defined as the duration of plasma irradiation; *t*_*r*_ is defined as the time between completion of the plasma irradiation process and injection of plasma-irradiated HBS; *V*_*p-p*_ represents the applied peak-to-peak voltage; *d*_*ele*_ is defined as the distance between the electrodes; and *L*_*g*_ is defined as the distance between the powered electrode and the edge of the glass tube. Typically, *V*_*p-p*_ = 5.0 kV; *L*_*g*_ = 23 mm; and *d*_*ele*_ = 38 mm. The flow rate of He gas (*f*) is regulated by a mass flow controller (MFC), and typically, *f* = 3 L/min.

Non-equilibrium APP can produce a variety of chemically reactive species due to its high reactivity. In particular, if helium (He) is used as the working gas, the presence of metastable helium (He^*^) in the plasma can greatly enhance the generation of reactive species such as ROS due to its high internal energy (~20 eV)^11^,^12^,^13^,^14^,^15^,^16^,^17^,^22^. Figure 1 schematically illustrates the experimental He-APP irradiation system and set-up for live-imaging of [Ca^2+^]_i_ after injection of the He-APP irradiated solution. In this system, He gas serves as the source gas, with its flow rate (***f***) through the dielectric tube regulated by a mass flow controller (MFC), and typically, ***f*** = 3 L/min. When the high-voltage (***V***_***p-p***_) power supply (with a frequency of 10 kHz) of this system is turned on, dielectric barrier discharge plasma is generated and flows out from the nozzle of the quartz glass tube (6-mm inner diameter), irradiating a 3-mL volume of live-imaging HEPES-buffered saline (HBS; containing 150 mM NaCl, 5 mM KCl, 2 mM CaCl_2_, 1 mM MgCl_2_, and 10 mM HEPES [pH adjusted to 7.4 with NaOH]), with or without 5.6 mM D-glucose. Typically, ***V***_***p-p***_ = 5.0 kV, ***L***_***g***_ = 23 mm, and ***d***_***ele***_ = 38 mm. The parameter ***t***_***i***_ is defined as the duration of plasma irradiation, and ***t***_***r***_ is defined as the time until injection of plasma-irradiated HBS after completion of the plasma irradiation process. During and after the addition of plasma-irradiated solution (indirect plasma irradiation), real-time changes in [Ca^2+^]_i_ are measured using a confocal microscope (FV1000, Olympus) with a fluo-4 AM calcium indicator (F-14201, Invitrogen).

## Results

### Production of hydrogen peroxide (H_2_O_2_) and hydroxyl (OH) radicals in solution by He-APP irradiation

**Figure 2:**
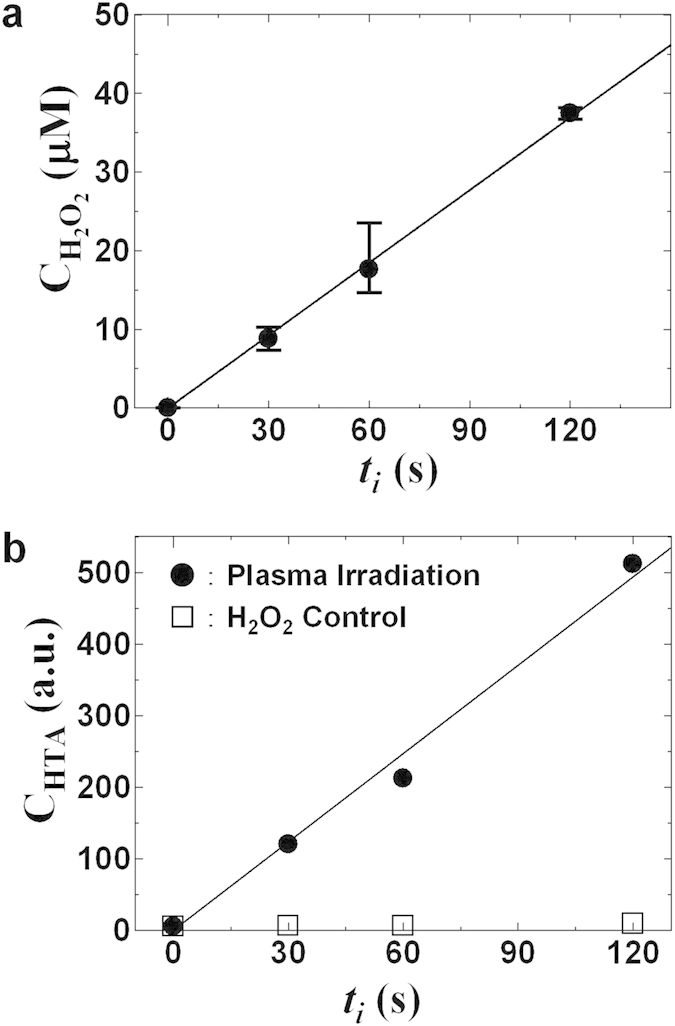
H_2_O_2_ and OH radicals are produced in HBS by He-APP irradiation. (**a**) H_2_O_2_ concentration (C_H2O2_) and (**b**) total production of OH radicals in plasma-irradiated HBS as a function of plasma irradiation time (***t***_***i***_). The OH radical can convert terephthalate anion (which is produced from terephthalic acid [TA]) to 2-hydroxyterephthalate ion (HTA) as a highly fluorescent material. The concentration of HTA (C_HTA_) indicates the total production of OH radicals in the solution. Although plasma irradiation of HBS resulted in H_2_O_2_ generation (e.g., 10 μM H_2_O_2_ for plasma irradiated for 30 s), the H_2_O_2_ control plot indicates that HTA is not produced simply by adding this level of H_2_O_2_ to HBS.

A wide variety of plasma-produced chemically reactive species are expected, and the specific species produced and their reactivity are expected to vary over time. In terms of their life span in the solution, these species can be classified as long-lived (life span on the order of hours or more), short-lived (life span on the order of minutes), or extremely short-lived (life span on the order of seconds or less). One of the long-lived products is H_2_O_2_, which can significantly impact biological responses. The H_2_O_2_ concentration (C_H2O2_) in the solution after plasma irradiation for ***t***_***i***_ was estimated using a colorimetric staining probe (WAK-H2O2, Kyoritsu Chemical-Check Laboratory). As shown in Fig. 2a, C_H2O2_ increased linearly with ***t***_***i***_. Only 2.9 μM H_2_O_2_ was generated in 3 mL of HBS after plasma irradiation for 10 s. This level of H_2_O_2_ reportedly causes minimal cytotoxicity and does not appreciably affect cellular proliferation^23^.

Total production of the extremely short-lived OH radical species in the solution after plasma irradiation for ***t***_***i***_ (also determined using chemical dosimetry based on terephthalic acid [TA]^24^) also increased linearly with ***t***_***i***_ (Fig. 2b). Although plasma irradiation resulted in a significant increase in the generation of OH radicals along with the generation of H_2_O_2_ (e.g., 10 μM H_2_O_2_ generated at ***t***_***i***_ = 30s) in the HBS, direct addition of this level of H_2_O_2_ to HBS (H_2_O_2_ control) failed to generate any detectable level of OH radicals. Therefore, the observed OH radicals in HBS were generated by He-APP irradiation. OH radicals are believed to play an important role in plasma-mediated biological responses targeted in plasma medicine due to their high reactivity and oxidation potential^1^,^2^,^3^,^4^,^25^,^26^. The presence of OH radicals in the solution therefore indicates that many different chemically reactive species are actually produced, with reactions involving OH radicals serving as potential triggers for inducing various biological responses after plasma irradiation of HBS. The production of OH radicals could not be mimicked by direct H_2_O_2_ administration. As time proceeds, the extremely short-lived chemically reactive species apparently rapidly break down into short-lived and long-lived species such as H_2_O_2_.

### Plasma-irradiated HBS elicited an increase in [Ca^2+^]_i_ in 3T3L1 fibroblasts

**Figure 3:**
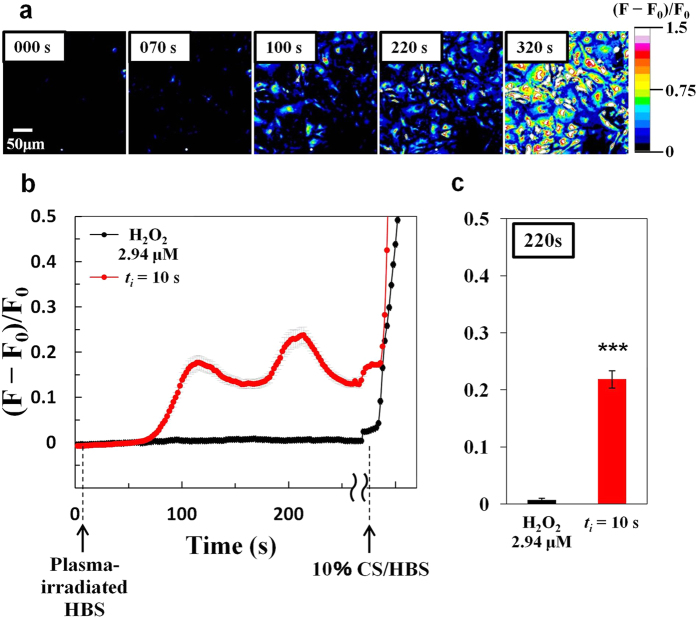
Increase in average [Ca^2+^]_i_ induced by administration of plasma-irradiated HBS to the culture. (**a**) Time-lapse images showing changes in [Ca^2+^]_i_ in 3T3-L1 cells stimulated with plasma-irradiated HBS (***t***_***i***_ = 10 s; ***t***_***r***_ = 30 s). (**b**) Time course of changes in average [Ca^2+^]_i_ and (c) [Ca^2+^]_i_ level after a 220-s lag period in 3T3-L1 cells stimulated with plasma-irradiated HBS (***t***_***i***_ = 10 s; ***t***_***r***_ = 30 s) (*red line*) and 2.94 μM H_2_O_2_ (*black line*) at indicated times (arrows indicate time points when each solution was injected). The HBS containing 10% calf serum (CS) [10% CS/HBS] is used as positive control for [Ca^2+^]_i_ increase. The mean values ± SE obtained from 40 cells (***t***_***i***_ = 10 s; ***t***_***r***_ = 30 s) and 38 cells (H_2_O_2_) are shown. Statistical analysis was performed with Mann-whitney u-test (***p < 0.001).

Administration of plasma-irradiated HBS (*t*_*i*_ = 10 s) to cells in culture resulted in gradual and sometimes oscillatory increases in [Ca^2+^]_i_ after a relatively long lag period (~70 s), whereas administration of naive HBS containing 10% calf serum (10% CS/HBS) as a positive control induced a rapid increase in [Ca^2+^]_i_ (Fig. 3a,b, *red line*, Supplementary Movie 1). In contrast, administration of HBS containing 2.9 μM H_2_O_2_ (H_2_O_2_ control) failed to induce any increase in [Ca^2+^]_i_ (Fig. 3b,c, *black line*). These results suggest that chemically reactive species other than H_2_O_2_ in the plasma-irradiated HBS induced the increase in [Ca^2+^]_i_, possibly ROS, which could have been generated in the HBS as one of the initial reaction products (e.g., OH radicals), as shown in Fig. 2b.

**Figure 4:**
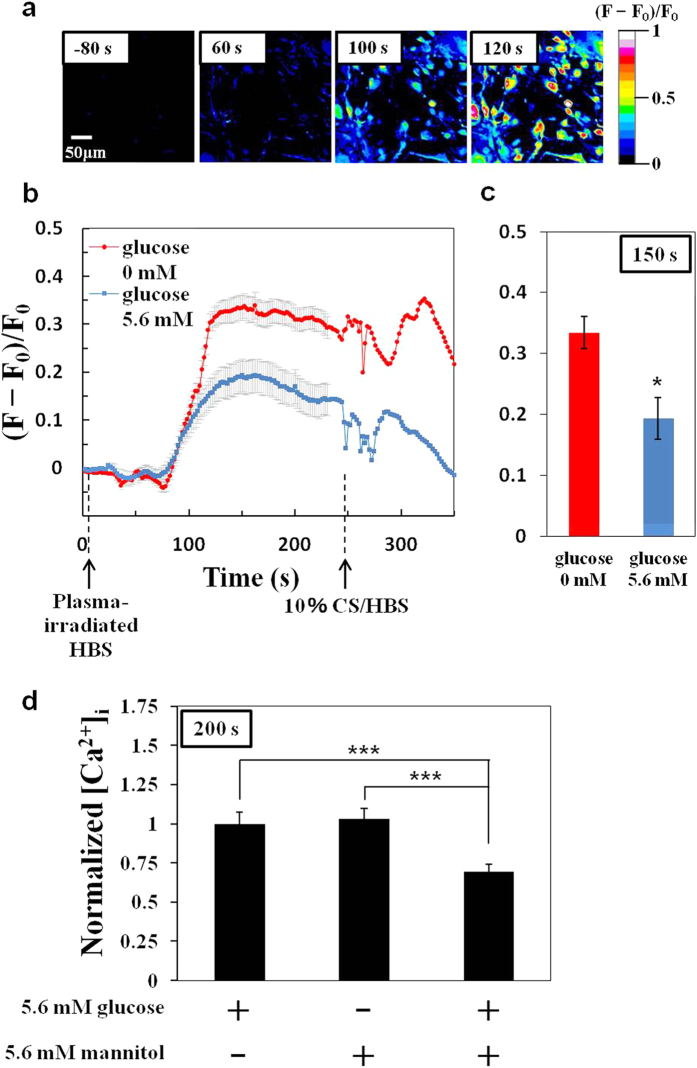
D-glucose and D-mannitol suppress the plasma-induced increase in [Ca^2+^]_i_. (**a**) Time-lapse images showing the change in [Ca^2+^]_i_ in 3T3-L1 cells stimulated with plasma-irradiated HBS without D-glucose (***t***_***i***_ = 10 s; ***t***_***r***_ = 30 s). (**b**) Time course of changes in average [Ca^2+^]_i_ and (**c**) [Ca^2+^]_i_ level after a 150-s lag period in 3T3-L1 cells stimulated with plasma-irradiated HBS in the absence (*red line*) and presence (*blue line*) of D-glucose (***t***_***i***_ = 10 s; ***t***_***r***_ = 30 s). The HBS containing 10% calf serum (CS) [10% CS/HBS] is used as positive control for [Ca^2+^]_i_ increase. The mean values ± SE obtained from 35 cells are shown. Statistical analysis was performed with Mann-whitney u-test (*p < 0.05). (**d**) Normalized [Ca^2+^]_i_ level after a 200-s lag period in 3T3-L1 cells stimulated with plasma-irradiated HBS [(5.6 mM glucose, 0 mM mannitol), (0 mM glucose, 5.6 mM mannitol), and (5.6 mM glucose, 5.6 mM mannitol)] for ***t***_***i***_ = 10 s; ***t***_***r***_ = 30 s. Each mean [Ca^2+^]_i_ was normalized at mean value in the sample of (5.6 mM glucose, 0 mM mannitol), and the mean values ± SE obtained from 48 cells (5.6 mM glucose, 0 mM mannitol), 50 cells (0 mM glucose, 5.6 mM mannitol), and 39 cells (5.6 mM glucose, 5.6 mM mannitol) are shown. Statistical analysis was performed with Steel-Dwass test for multiple comparisons (***p < 0.001).

To clarify the possible involvement of OH radicals in the increase in [Ca^2+^]_i_ induced by plasma-irradiated HBS, we compared [Ca^2+^]_i_ responses in the absence and presence of 5.6 mM D-glucose (an OH radical scavenger) in the HBS (Fig. 4b,c). In the case of glucose-free HBS (Fig. 4a), [Ca^2+^]_i_ was significantly higher compared with HBS containing 5.6 mM glucose. On the other hand, because D-glucose serves not only as an OH radical scavenger but also as the major energy source for living cells, the additional verification is necessary. We therefore examined effect of D-mannitol as another OH radical scavenger^26^, displaying less-permeable/less-metabolizable property, and found that 5.6 mM D-mannitol similarly suppressed the plasma-induced [Ca^2+^]_i_ increase (Fig. 4d). These results suggest that the effect of glucose metabolism would be minimal for at least a few minutes of this live-cell imaging. In the presence of 5.6 mM D-glucose, further addition of 5.6 mM D-mannitol additively suppressed the [Ca^2+^]_i_ level, strongly suggesting that ROS produced by an OH radical–initiated reaction are responsible for the increase in [Ca^2+^]_i_ elicited by the exposure of cells to plasma-irradiated HBS.

Based upon these observations, we conclude that ROS in plasma-irradiated HBS, rather than H_2_O_2_ (~2.9 μM), plays the predominant role in triggering changes in [Ca^2+^]_i_, although higher concentrations of H_2_O_2_ (30 μM) have been shown to induce increases in [Ca^2+^]_i_ mediated through TRPA1 channels in other cell types^27^. It should be noted that the [Ca^2+^]_i_ responses induced by plasma-irradiated HBS were not observed in the present study in MCF-7 human breast adenocarcinoma cells (data not shown).

### Ruthenium red and SKF 96365 strongly suppressed plasma-induced [Ca^2+^]_i_ changes associated with calcium influx

**Figure 5:**
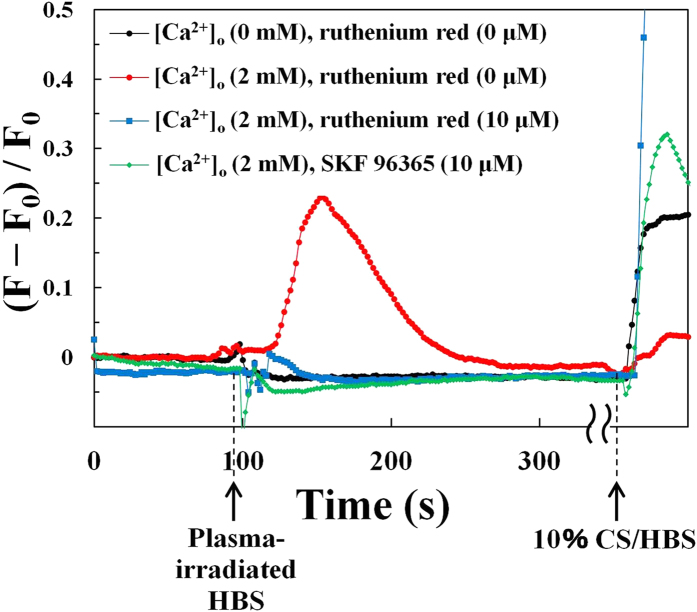
Ruthenium red (10 μM) and SKF 96365 (10 μM) strongly suppresses plasma-induced changes in [Ca^2+^]_i_ associated with calcium influx (*t*_*i*_ = 30 s; *t*_*r*_ = 30 s). Time course of change in [Ca^2+^]_i_ as determined from the average pixel intensity in 3T3-L1 cells stimulated with plasma-irradiated HBS (0 mM [Ca^2+^], 0 μM ruthenium red) [*black line*], (2 mM [Ca^2+^], 0 μM ruthenium red) [*red line*], (2 mM [Ca^2+^], 10 μM ruthenium red) [*blue line*], and 2 mM [Ca^2+^], 10 μM SKF 96365) [*green line*]. To the sample of (2 mM [Ca^2+^], 10 μM ruthenium red), 10 μM ruthenium red/HBS (not irradiated) is added at −240 s (340 s before the addition of plasma-irradiated solution) and have no effect on [Ca^2+^]_i_. To the sample of (2 mM [Ca^2+^], 10 μM SKF 96365), 10 μM SKF 96365/HBS (not irradiated) is added at −180 s (280 s before the addition of plasma-irradiated solution) and induce the temporary- and small- [Ca^2+^]_i_, but the [Ca^2+^]_i_ returns to the original level within 60 s. The HBS containing 10% calf serum (CS) [10% CS/HBS] is used as positive control for [Ca^2+^]_i_ increase.

Although we observed a sharp [Ca^2+^]_i_ spike after addition of calcium-containing plasma-irradiated HBS ([Ca^2+^] = 2 mM; ***t***_***i***_ = 30 s), the characteristic [Ca^2+^]_i_ spike was suppressed in cells treated with nominally calcium-free plasma-irradiated HBS ([Ca^2+^] = 0 mM) (Fig. 5, *black line*). In addition, the [Ca^2+^]_i_ responses evoked by plasma-irradiated HBS were completely blunted in the presence of ruthenium red (RR) and SKF-96365 (SKF), noncompetitive pan inhibitors of multiple TRP channels^28^,^29^,^30^, whereas 10% CS/HBS as a control induced significant [Ca^2+^]_i_ responses even in the presence of RR (Fig. 5, *blue line*) and SKF (Fig. 5, *green line*). Thus, these results indicate that the observed [Ca^2+^]_i_ increase following administration of plasma-irradiated HBS was mediated *via* the influx of extracellular Ca^2+^ and could not be attributed to calcium release from the ER. These results also strongly suggest that TRP channel(s) are involved in [Ca^2+^]_i_ responses elicited by chemically reactive species produced in plasma-irradiated HBS.

### Single-cell analyses of [Ca^2+^]_i_ responses induced by short-lived products in plasma-irradiated HBS

**Figure 6:**
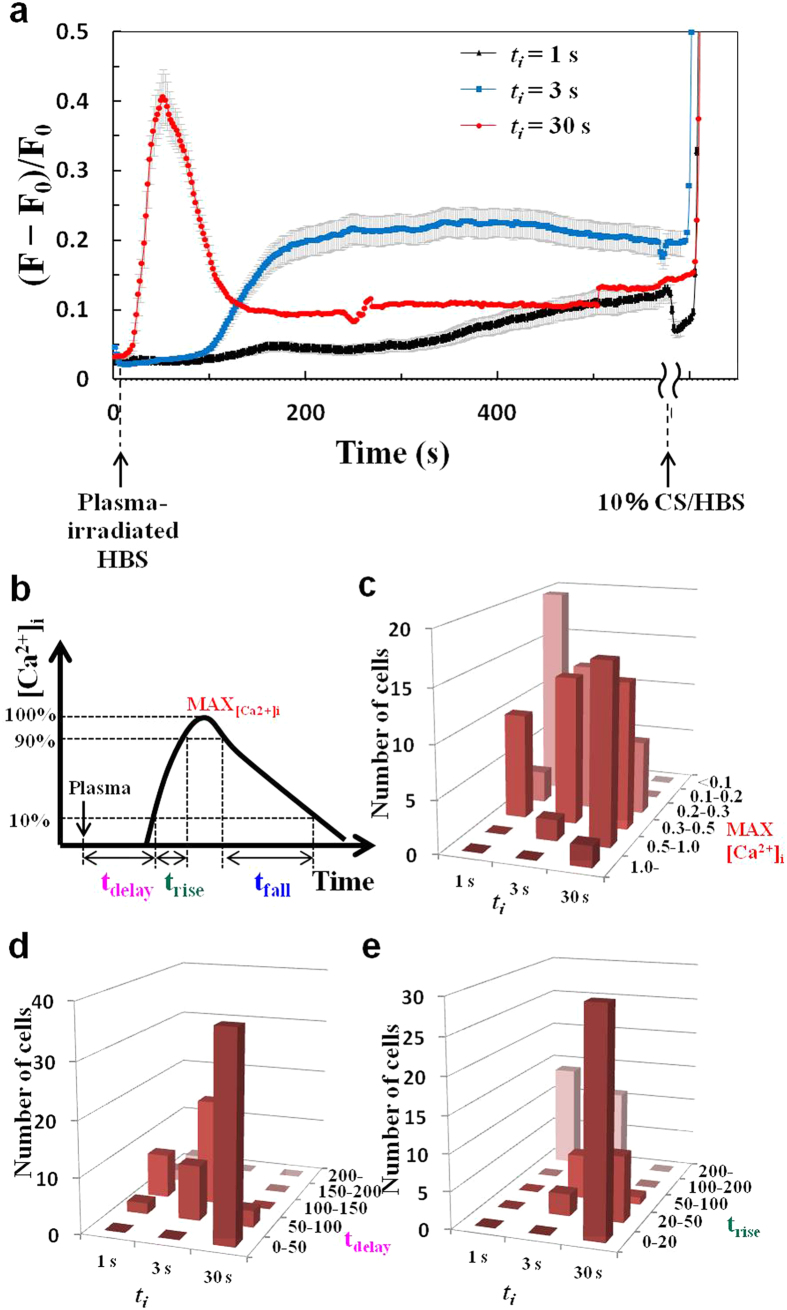
The nature of the [Ca^2+^]_i_ response induced by plasma-irradiated HBS is defined by the concentration of chemically reactive species in a manner dependent on plasma irradiation time (*t*_*i*_). Single-cell analyses of the [Ca^2+^]_i_ response elicited by plasma-irradiated HBS at varying *t*_*i*_ (*t*_*r*_ = 30s). (**a**) Time course of changes in the average [Ca^2+^]_i_ of 3T3-L1 cells stimulated with plasma-irradiated HBS at *t*_*i*_ = 1 s (*black line*), 3s (*blue line*), and 30s (*red line*). The HBS containing 10% calf serum (CS) [10% CS/HBS] is used as positive control for [Ca^2+^]_i_ increase. The mean values ± SE obtained from 40 cells are shown. The mean MAX_[Ca2+]i_ ± SE were 0.20 ± 0.02 (*t*_*i*_ = 1 s, p < 0.05 versus *t*_*i*_ = 3 s and p < 0.001 versus *t*_*i*_ = 30 s), 0.27 ± 0.02 (*t*_*i*_ = 3 s, p < 0.001 versus *t*_*i*_ = 30 s), and 0.52 ± 0.04 (*t*_*i*_ = 30 s). Statistical analysis was performed with Steel-Dwass test for multiple comparisons. (**b**) Definition of the parameters MAX_[Ca2+]i_, t_delay_, t_rise_, and t_fall_ for each cell. Histograms showing the distributions of (**c**) MAX_[Ca2+]i_, (**d**) t_delay_, and (**e**) t_rise_ for *t*_*i*_ = 1, 3, and 30 s.

To explore the underlying mechanism of the [Ca^2+^]_i_ responses elicited by plasma-irradiated HBS, HBS plasma irradiated for 1, 3, or 30 s was administrated to cells, and [Ca^2+^]_i_ responses of individual cells were then carefully analyzed. Whereas administration of plasma-irradiated HBS (***t***_***i***_ = 10 s) resulted in a significant increase in [Ca^2+^]_i_ after a 70-s lag period, as shown in Figs 3 and 4, plasma-irradiated HBS (***t***_***i***_ = 30 s) induced large, sharp spikes in [Ca^2+^]_i_ (lasting for ~100 s), followed by prolonged periods (at least 10 min) of lower [Ca^2+^]_i_, although these lower levels were still significantly higher than the control (Fig. 6a, *red line*). As expected, (***t***_***i***_ = 3 s) plasma-irradiated HBS required a much longer lag period (~100 s) before commencement of the significant increase in [Ca^2+^]_i_, although in this case, the [Ca^2+^]_i_ peak was not as sharp as that observed with administration of (***t***_***i***_ = 30 s) plasma-irradiated HBS (Fig. 6a, *blue line*).

As the profiles of [Ca^2+^]_i_ responses elicited by administration of plasma-irradiated HBS were highly complex and appeared to involve a variety of ROS (which then partially/gradually degenerated) generated in the HBS by differing intensities (periods) of plasma irradiation, we examined the [Ca^2+^]_i_ responses at the single-cell level based on analyses of four parameters (MAX_[Ca2+]i_, t_delay_, t_rise_, t_fall_), as shown Fig. 6b. MAX_[Ca2+]i_ represents the maximum value of [Ca^2+^]_i_ after addition of plasma-irradiated HBS; t_delay_ represents the time required to reach a level of 10% of the increase in [Ca^2+^]_i_ after exposure to plasma-irradiated HBS; t_rise_ represents the interval between the time the 10% level in the increase in [Ca^2+^]_i_ is attained and the time the 90% level in the increase in [Ca^2+^]_i_ is attained; and t_fall_ represents the interval between the time the 90% level in the increase in [Ca^2+^]_i_ is attained and the time [Ca^2+^]_i_ returns to the 10% level. Each of these parameters was determined for each cell examined, and the results are depicted as a function of ***t***_***i***_ in Fig. 6c, d, e. As ***t***_***i***_increased, more cells exhibited a higher MAX_[Ca2+]i_, and the mean/median values for MAX_[Ca2+]i_ increased (Fig. 6c). In addition, both t_delay_ and t_rise_ declined with increasing ***t***_***i***_ (Fig. 6d,e). Thus, the nature of the [Ca^2+^]_i_ response induced by administration of plasma-irradiated HBS is apparently defined by the concentrations of chemically reactive species that are generated by plasma irradiation, in a manner dependent on ***t***_***i***_.

**Figure 7:**
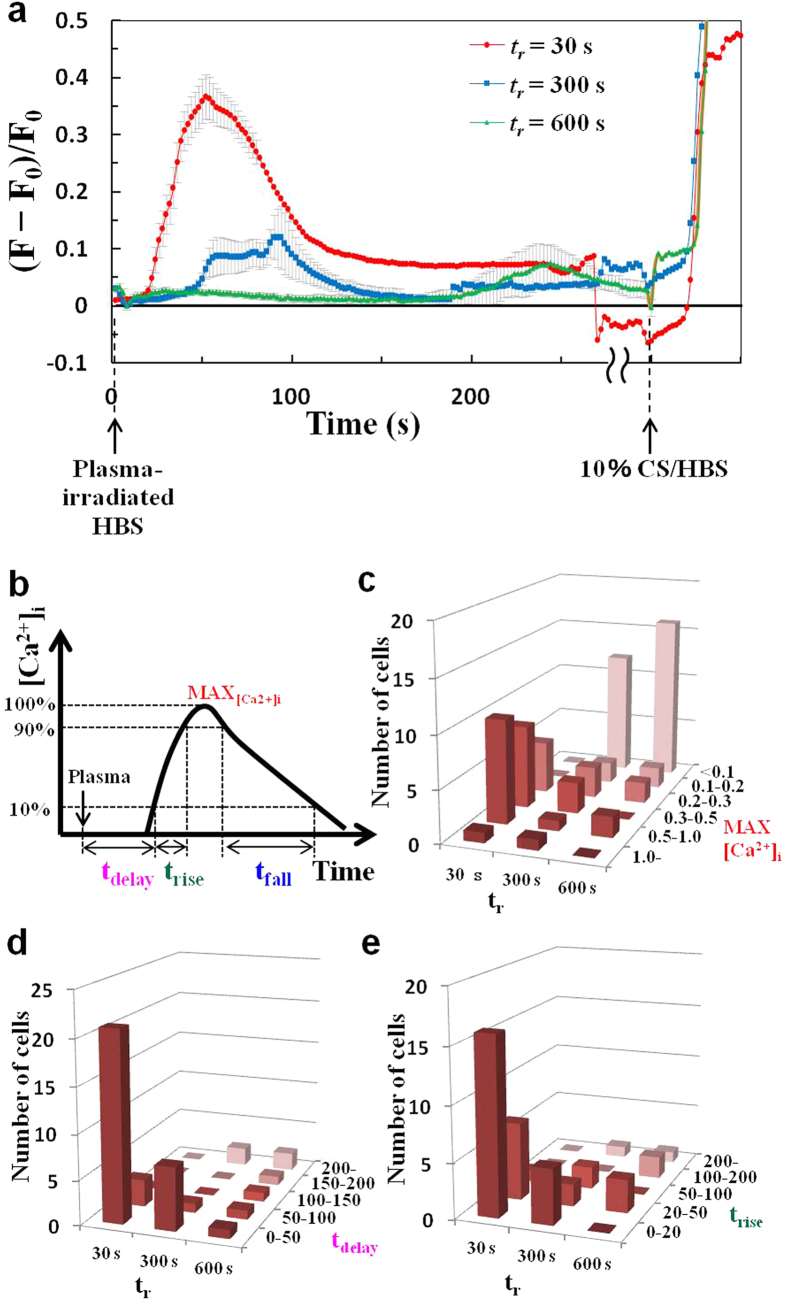
Relationship between retention time (*t*_*r*_) of plasma-irradiated HBS and its potency for evoking [Ca^2+^]_i_ responses. Single-cell analyses of the [Ca^2+^]_i_ response evoked by plasma-irradiated HBS at varying ***t***_***r***_ (***t***_***i***_ = 30 s). (**a**) Time course of changes in the average [Ca^2+^]_i_ of 3T3-L1 cells stimulated with plasma-irradiated HBS at ***t***_***r***_ = 30 s (*red line*), 300 s (*blue line*), and 600 s (*green line*). The HBS containing 10% calf serum (CS) [10% CS/HBS] is used as positive control for [Ca^2+^]_i_ increase. The mean values ± SE obtained from 23 cells are shown. The mean MAX_[Ca2+]i_ ± SE were 0.50 ± 0.05 (***t***_***r***_ = 30 s, p < 0.001 versus ***t***_***r***_ = 300 s and ***t***_***r***_ = 600 s), 0.20 ± 0.06 (***t***_***r***_ = 300 s), and 0.14 ± 0.04 (***t***_***r***_ = 600 s). Statistical analysis was performed with Steel-Dwass test for multiple comparisons. (**b**) Definition of the parameters MAX_[Ca2+]i_, t_delay_, t_rise_ and t_fall_ for each cell. Histograms showing the distributions of (**c**) MAX_[Ca2+]i_, (**d**) t_delay_, and (**e**) t_rise_ for ***t***_***r***_ = 30, 300, and 600 s.

**Figure 8:**
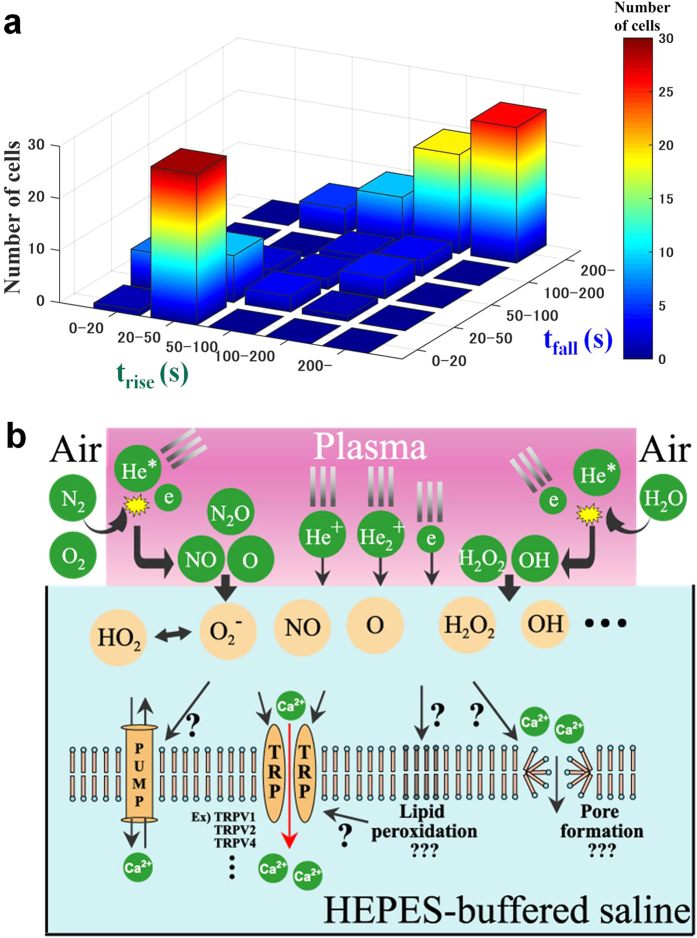
Plasma-produced short-lived reactive species in HBS regulate not only calcium influx but also calcium efflux. (**a**) 3D (color map) histogram of t_rise_ and t_fall_ for all conditions. (**b**) Model of calcium influx mediated by short-lived species in plasma-irradiated HBS administered to 3T3-L1 cells.

Most of the chemically reactive species generated by plasma irradiation are assumed to be short-lived and to dissipate rapidly over time. Therefore, we next investigated the amount of time plasma-irradiated HBS retains its potency for evoking [Ca^2+^]_i_ responses (Fig. 7). Plasma-irradiated HBS was administrated to cells at 30, 300, or 600 s after irradiation (***t***_***i***_ = 30 s), and the [Ca^2+^]_i_ response was then carefully analyzed. As expected, the [Ca^2+^]_i_ responses changed dramatically as the retention time after plasma-irradiation (***t***_***r***_) increased. As ***t***_***r***_ increased, the increase in [Ca^2+^]_i_ was lower, and the time required to reach the maximal value increased (i.e., the response was slower). In addition, single-cell analyses of [Ca^2+^]_i_ responses in the same manner as described above showed that most of cells exhibited lower MAX_[Ca2+]i_ values with increasing ***t***_***r***_, although some cells maintained a high MAX_[Ca2+]i_. Similarly, although both t_delay_ and t_rise_ tended to be longer for most of the cells, some cells maintained short t_delay_ and t_rise_. Taken together, these observations demonstrate that the chemically reactive species in plasma-irradiated HBS that exhibit the highest potency in evoking [Ca^2+^]_i_ responses have a life span of the order of several minutes. Furthermore, the results of single-cell analyses suggest that differences in results between retention experiments is due to ***t***_***r***_-dependent deactivation-associated changes in the composition of the chemically reactive species in plasma-irradiated HBS retained for different periods before administration. The above results indicate that the nature of the [Ca^2+^]_i_ response is strongly influenced by the concentration and composition of chemically reactive species in the plasma-irradiated HBS, which apparently have life spans on the order of several minutes. In addition, these results indicate that t_rise_ is strongly correlated with t_fall_ (Fig. 8a); that is, a sharper rise in [Ca^2+^]_i_ tends to cause a sharper fall in [Ca^2+^]_i_.

## Discussion

Despite the promising potential of plasma medicine based on non-equilibrium APP technology, a crucial issue remains unresolved. That is, how do cells decipher and respond to the wide array of highly complex and interrelated stimuli evoked by APP irradiation, including irradiation with UV rays, ROS, RNS, electric fields, and shock waves^1^,^2^,^3^,^4^? In an attempt to dissect these complex stimuli that directly and/or indirectly affect cellular functions, we focused on the biologically active elements generated in the medium (a biological buffer) after non-equilibrium He-APP irradiation by monitoring intracellular Ca^2+^ dynamics. A key finding of the present study is that plasma-irradiated HBS contains chemically reactive species that can induce physiologically relevant increases in [Ca^2+^]_i_ by triggering Ca^2+^ influx (Figs 3 and 4) through RR- and SKF-sensitive Ca^2+^-permeable channel(s) (Fig. 5), possibly a member(s) of the TRP channel family^28^,^29^,^30^. Based upon our detailed analyses of the [Ca^2+^]_i_ responses in 3T3-L1 fibroblasts (Figs 3, 4, 5, 6, 7), we can conclude that plasma irradiation creates very potent, but relatively short-lived, chemically reactive species that trigger Ca^2+^ influx (Fig. 8b). The potency of these species is not attributable to only H_2_O_2_, which is one of the end products of plasma irradiation (Fig. 3). Although elucidating the nature of the plasma-generated bioactive species and clarifying the identity of Ca^2+^-permeable channel(s) responsible for these [Ca^2+^]_i_ responses will be challenging, our novel findings provide important insights that will enhance our understanding of plasma-mediated biological responses, at least with respect to the associated constituents, consisting presumably of multiple reactive species uniquely created in the medium by non-equilibrium He-APP irradiation.

In this study, we utilized non-equilibrium APP, which allows for a strong non-equilibrium chemical reaction of O_2_ and N_2_ in ambient air, resulting in the efficient generation of chemically reactive species including ozone/O_3_^14^, nitric oxide/NO^14^, atomic oxygen/O^15^, and OH radicals^15^ as well as their unclassified derivatives at a specific constituent ratio. Some of these chemically reactive species reportedly exert both beneficial and detrimental biological effects on cells, mediated by various ROS-sensing mechanisms^3^,^31^. Our results provide compelling evidence that the [Ca^2+^]_i_ responses evoked by plasma-irradiated HBS involve RR- and SKF-sensitive Ca^2+^-permeable channel(s) (Fig. 5), presumably members of TRP channel. Indeed, it is increasingly apparent that a subclass of TRP channels function as chemosensors for detecting various reactive species, such as ROS and RNS^21^,^32^. For example, TRPA1 is reportedly activated by H_2_O_2_ (~30 μM), S-nitroso-N-acetyl-DL-penicillamine (SNAP), which is an NO donor, as well as by other inflammatory mediators. These [Ca^2+^]_i_ responses are blunted by substitution of the redox-sensitive cysteine residues in TRPA1, which can be modified by oxidative covalent reaction (e.g., S-nitrosylation)^33^. In addition to TRPA1, the channel activity of many other TRPs (TPRV1, V2, V3, V4, and C5) has been shown to be regulated in a redox-sensitive manner with different electron acceptor (oxidation) capacities^34^. Furthermore, oxidative stress reportedly led to a phenotypic shift in Ca^2+^ mobilization from an oscillatory to a sustained elevated pattern via calcium release–activated calcium (CRAC)–mediated capacitive Ca^2+^ entry through Orai channel activated by stromal interaction molecule 1 (STIM1) on ER, which is also possibly involving TRP channel(s)^35^,^36^. While observed Ca^2+^ influx is clearly triggered through RR- and SKF-sensitive Ca^2+^-permeable channels, possibly TRP channel(s), secondary calcium dynamics (e.g. oscillatory increases in Fig. 3) might be related to STIM1 activation. In addition, it has been reported that plasma-produced species can directly peroxidate lipids^37^,^38^ and that TRP channels are activated by oxidized lipids^39^, and thereby, it is also possible that plasma-produced species may activate TRP channels through lipid peroxidation. Considering these highly sensitive redox-responsive characteristics of TRP channels in light of our results (Figs 3, 4, 5, 6, 7), it is highly likely that the [Ca^2+^]_i_ responses in 3T3L1 cells are mediated via TRP channel(s)^40^, which are activated by relatively short-lived ROS (deactivated within approximately 10 min) rather than extremely short-lived (OH radicals) or long-lived (H_2_O_2_) reactive species. As research indicates that TRP channels are promising drug targets^41^, plasma-irradiated solutions could be utilized in medical applications, though the interactions between TRP channels and plasma-induced chemically reactive species need to be further elucidated.

Plasma medicine is a rapidly emerging field that combines plasma physics, life sciences, and clinical medicine with the goal of developing therapeutic applications for physical plasma^1^,^2^,^3^,^4^. The biological responses to direct cold plasma and plasma-irradiated solutions have been the subject of considerable recent research. The sensitivity of cells to chemically reactive species is thought to depend on the unique properties of each cell type. However, many previous reports on the biological responses to plasma-irradiated solutions may actually involve calcium influx through TRP channels, as reported in this paper. We anticipate that our results will contribute to further progress in the field of plasma medicine.

## Methods

### Cell culture

Mouse 3T3-L1 fibroblasts (ATCC CL-173) and human breast adenocarcinoma MCF-7 cells (RBRC-RCB1904) were obtained from ATCC and RIKEN BioResource Center, respectively. 3T3-L1 fibroblasts were maintained in DMEM supplemented with 10% calf serum (CS), 100 U/mL penicillin, and 100 μg/mL streptomycin (growth medium) at 37 °C in an 8% CO_2_ atmosphere. For observation, cells were re-plated onto glass-bottomed recording chambers and cultured for 1–3 additional days.

### Indirect plasma irradiation

HBS without cells was directly irradiated with APP. The applied voltage and frequency were 5.0 kV and 10 kHz, respectively. The helium gas flow rate was 3 L/min. After a prescribed time (***t***_***r***_) after plasma irradiation, the plasma-irradiated HBS was added to cells.

### Measurement of changes in [Ca^2+^]_i_

The acetoxymethyl (AM) ester form of fluo-4 (F-14201, Invitrogen) was dissolved in dimethyl sulfoxide (DMSO) at 5 mM. 3T3-L1 cells in a glass-bottomed recording chamber (thickness, 0.15–0.18 mm; Matsunami-glass) were incubated in serum-free DMEM-HG containing 5 μM fluo 4-AM and 0.03% Cremophor-EL (C5135, Sigma-Aldrich) for 30 min at 37 °C. The cells were then washed with HBS containing 5.6 mM D-glucose (Figs 2, 3, 4, 5, 6, 7) or without D-glucose (Fig. 4). Images were acquired every 2 s using a confocal microscope (FV1000, Olympus). Changes in [Ca^2+^]_i_ were expressed as (F − F_0_)/F_0_, where F and F_0_ represent the fluorescence intensity of fluo 4 and the averaged fluorescence intensity of the dye before stimulation with plasma-irradiated HBS, respectively.

## Additional Information

**How to cite this article**: Sasaki, S. *et al.* Calcium influx through TRP channels induced by short-lived reactive species in plasma-irradiated solution. *Sci. Rep.* **6**, 25728; doi: 10.1038/srep25728 (2016).

## Supplementary Material

Supplementary Movie 1

Supplementary Information
