# Supplementary material for: Calcium influx through TRP channels induced by short-lived reactive species in plasma-irradiated solution
Source: Sci Rep. 2016 May 12;6:25728. doi: 10.1038/srep25728 (PMC4864414; doi:10.1038/srep25728)
Supplement: Supplementary Information [file srep25728-s2.pdf]

Calcium influx through TRP channels induced by short-lived reactive species in plasma-irradiated solution

Shota Sasaki[1,*], Makoto Kanzaki[2], and Toshiro Kaneko[1]

[1]*Department of Electronic Engineering, Tohoku University,*

6-6-05 Aoba, Aramaki, Aoba-ku, Sendai 980-8579, Japan

[2]*Department of Biomedical Engineering, Tohoku University,*

6-6-04 Aoba, Aramaki, Aoba-ku, Sendai 980-8579, Japan

** e-mail address: sasaki12@ecei.tohoku.ac.jp*

Supplementary Movie

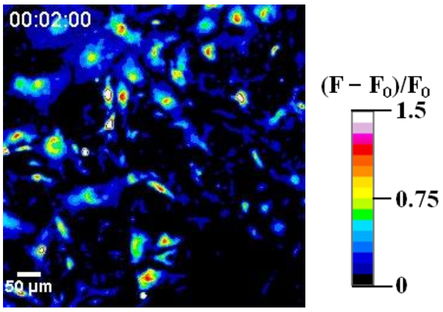

Supplementary Movie 1. Time-lapse movie showing changes in $[Ca^{2+}]_i$ in 3T3-L1 cells stimulated with plasma-irradiated HBS ($\boldsymbol{t_i}$ = 10 s). (The plasma- plasma-irradiated solution was injected at 0 s)
